# Experimental and numerical data from stub-column, short beam-column and long beam-column tests on the local and global buckling behavior of RHS/SHS under N–M interaction

**DOI:** 10.1016/j.dib.2024.110162

**Published:** 2024-02-07

**Authors:** Andreas Müller, Andrea Toffolon, Andreas Taras

**Affiliations:** a ETH Zurich, D-BAUG, Institute of Structural Engineering, Zurich, Switzerland; b Bundeswehr University Munich, Chair of Steel Structures, Institute of Structural Engineering, Germany

**Keywords:** Local and global buckling, GMNIA, Hollosstab, 3D scanning, Reverse engineering, High-strength steel

## Abstract

The presented data is based on investigations carried out in the framework of the European RFCS (Research Fund for Coal and Steel) funded project HOLLOSSTAB (2016-2019). The campaign's overall goal is presented in more detail in [1] and [2]. The experiments were performed in the Structural Laboratory at the Bundeswehr University Munich to investigate the cross-section behavior of cold-formed square and rectangular hollow sections (SHS and RHS). Two grades of mild and high-strength steel (S355 and S500) and seven section sizes were examined. The profiles cover all four cross-section classes according to EN1993-1-1 [3]. Monotonic stub column, short beam, and long-beam column tests were performed to investigate the load-bearing capacity. The outputs were load-deformation curves for each specimen. The experimental tests were accomplished by digital image correlation (DIC) to obtain an overview of the full deformation field in the specimens. Recalculations with advanced FE-shell simulations, based on scanned specimen geometries (spatial 3D point clouds) and nonlinear material models obtained from tensile coupon tests, were modeled to reproduce the real behavior obtained during the tests.

Specifications Table

**Table:** 

Subject	Engineering	
Specific subject area	Structural Engineering	
Data format	Filtered data:	CSV files with load-deformation information CSV files with nodal point coordinates (3D point clouds) STP files of model (scanned specimen) geometry
	Analyzed data:	Excel files with load-deformation output INP files of numerical models Jpeg files of load-deformation curves
Type of data	Tables Graphs and Figures 3D geometry models Abaqus input files	
Data collection	Experimental test data was obtained from stub column, short beam-column, as well as long beam-column tests carried out on a testing machine built by MFL. Type UPS 1000V. The vertical load was transferred through an actuator with a maximum compression force of 10MN. A hydraulic load cell was used for measuring the actuator force with a pressure transducer of type HBM P3MB-350bar. Digital image correlation (DIC) was used to capture deformations and rotations of the specimen surface and of the test rig. The outer surface of the specimens was scanned using a 3D digitizing system by Carl Zeiss Optotechnic GmbH. The obtained special point clouds were used for the assessment of local and global imperfections. Numerical simulations were performed using the commercially available software Abaqus. Each shell finite element model is based on the scanned spatial point cloud geometry, taking into account the real specimen geometry. Corresponding material models were derived from tensile coupon test. A standard hydraulic testing rig, produced by Zwick-Röll, was used to perform the experiments.	
Data source location	Chair of Steel Structures, Bundeswehr University Munich, Germany, Europe	
Data accessibility	Repository name: Hollosstab_SHS_RHS_Data Data identification number: https://doi.org/10.3929/ethz-b-000639467 Direct URL to data: https://www.research-collection.ethz.ch/handle/20.500.11850/639467	
Related research article	A. Toffolon, X. Meng, A. Taras, and L. Gardner, ‘The generalized slenderness-based resistance method for the design of SHS and RHS’, Steel Constr., vol. 12, no. 4, pp. 327–341, 2019. https://doi.org/10.1002/stco.201900036	

## 1 Value of the Data

• The presented data is based on selected results from the RFCS-funded research project HOLLOSSTAB (grant Nr. RFCS-2015-709892). The experimental and numerical results can be used to verify and validate own numerical models.
• The data benefits researchers and structural engineers from practice dealing with local instability problems in thin-walled RHS/SHS members, made from mild or high-strength steel.
• The experimental data can be taken for the assessment and interpretation of own test results or methodical approaches. Further, the numerical data can be reused in own simulations using the provided Abaqus input files with the information of the real geometry. Further, the 3D point cloud data of the scanned specimens can be taken for the interpretation of local and global imperfections (.txt file) or as the input geometry in own numerical simulations (.stp file).
• Since the point clouds are already optimized, i.e. cleaned from outliers and centered according to the Cartesian coordinate system, the provided data can directly be used for own investigations, i.e. reverse engineering, computer vision, automatic recognition of profiles and members.

## 2 Data Description

The presented data sets are based on monotonic stub column and short beam tests performed to investigate the cross-section behavior, i.e. the local buckling instability phenomena of RHS/SHS profiles made from cold-formed mild and high-strength steel and different N-M interactions. Therefore, full scale test are sampled into 5 categories, depending on the load case and the length of the used profiles. The test designation was chosen according to the cross-section geometry and eccentricity parameters “T1–T5”, as well as the steel grade. An exemplary designation is provided as follows:

• RHS profiles “Profile-type_height_width_thickness_category_steel grade”
  ○ Example: RHS_300_150_6_T1_S355
• SHS profiles “Profile-type_height_thickness_category_steel grade”.
  ○ Example: SHS_140_4_T1_S355

This naming retains for all provided files the same.

Data from experimental results is provided in the folder “Test_Data”. This folder contains individual CSV files of the load-deformation results for all profiles from Table 1. The data within each CSV file is represented by three columns described in the following:

• Stroke_Aramis_[mm]: Measured deformation from digital image correlation (DIC). This deformation is used as a reference to the applied deformation by the actuator in the test setup.
• Stroke_rig_sensor_corr_[mm]: Corrected applied deformation during the test. This deformation is already pre-processed excluding the test setup stiffness.
• Force_rig_senor_[kN]: Measured force by the hydraulic load cell.

**Table 1:** Summary of tested RHS and SHS sections at Bundeswehr University Munich; dimensions according to EN10219-2 [4].

Profile	Top eccentricity [mm]	Bottom eccentricity [mm]	Length [mm]	Steel grade
RHS 300 × 150 × 6_T1	0	0	800	S355
RHS 300 × 150 × 6_T2	17.9	0	800	S355
RHS 300 × 150 × 6_T3	57	57	800	S355
RHS 300 × 150 × 6_T4	297	297	800	S355
RHS 300 × 150 × 6_T5	57	57	2000	S355
RHS 300 × 150 × 8_T1	0	0	800	S355
RHS 300 × 150 × 8_T2	17.4	0	800	S355
RHS 300 × 150 × 8_T3	57	57	800	S355
RHS 300 × 150 × 8_T4	232	232	800	S355
SHS 140 × 4_T1	0	0	800	S355
SHS 140 × 4_T2	14.6	0	800	S355
SHS 140 × 4_T3	137	137	800	S355
SHS 140 × 4_T4	312	312	800	S355
SHS 140 × 4_T5	137	137	2000	S355
SHS 200 × 5_T1	0	0	800	S355
SHS 200 × 5_T2	20.9	0	800	S355
SHS 200 × 5_T3	107	107	800	S355
SHS 200 × 5_T4	457	457	800	S355
SHS 200 × 8_T1	0	0	800	S355
SHS 200 × 8_T2	20.9	0	800	S355
SHS 200 × 8_T3	107	107	800	S355
SHS 200 × 8_T4	457	457	800	S355
SHS 200 × 4_T1	0	0	800	S550
SHS 200 × 4_T2	63.0	0	800	S550
SHS 200 × 4_T3	107	107	800	S550
SHS 200 × 4_T4	457	457	800	S550
SHS 200 × 4_T5	107	107	2000	S550
SHS 200 × 5_T1	0	0	800	S550
SHS 200 × 5_T2	63.9	0	800	S550
SHS 200 × 5_T3	107	107	800	S550
SHS 200 × 5_T4	457	457	800	S550
SHS 200 × 5_T5	107	107	2000	S550

All specimens were 3D scanned before performing the full-scale test. The special point cloud data is collected and provided in the folder “Scan_Data”. This folder contains CSV files with the coordinates of the scanned specimens and STP files, which can be used in own numerical simulations as an input for the initial “real” 3D geometry. The data within each CSV file is represented by eleven columns described as follows:

• Row index
• Referenzpos. X: Reference position of the perfect geometry in x-direction in [mm]
• Referenzpos. Y: Reference position of the perfect geometry in y-direction in [mm]
• Referenzpos. Z: Reference position of the perfect geometry in z-direction in [mm]
• Gemessene Pos. X: Measured position of the scanned geometry in x-direction in [mm]
• Gemessene Pos. Y: Measured position of the scanned geometry in y-direction in [mm]
• Gemessene Pos. Z: Measured position of the scanned geometry in z-direction in [mm]
• Achsabw. X: Difference between Referenzpos. X and Gemessene Pos. X in [mm]
• Achsabw. Y: Difference between Referenzpos. Y and Gemessene Pos. Y in [mm]
• Achsabw. Z: Difference between Referenzpos. Z and Gemessene Pos. Z in [mm]
• Abweichung: Absolut difference in space

Geometrically and materially nonlinear shell-based simulations with imperfections were performed in Abaqus, including the “real” scanned geometry (GMNIA-Meas). The data is collected in the folder “Simulation_Data”. This folder contains Excel files with load-deformation data from GMNIA-Meas simulations and corresponding Abaqus input files, which can directly be used in own simulations. The data within each Excel file is represented by two columns, described as follows:

• Deformation [mm]: The deformation of the reference point at the top, the
• Load [N]: The load at the bottom reference point

Further, each Abaqus input (INP) file contains the geometry, material model and boundary conditions to replicate the numerical simulation actual test. The used nonlinear material model is based on tensile coupon test and is directly implemented using the true stress-strain relation.

A compilation of load-deformation diagrams and corresponding images from simulations and experiments is compiled in the folder “Comparison_Plots”. Two JPEG files for each specimen provide the overall information. The designation corresponds to the description above, with the exception of an addition of “1” or “2” at the end of each file name. The first (1) always shows the evaluation of the load-deformation curves, and the second (2) images of the buckled specimen from simulation, test, and DIC recording.

**Fig. 1:**
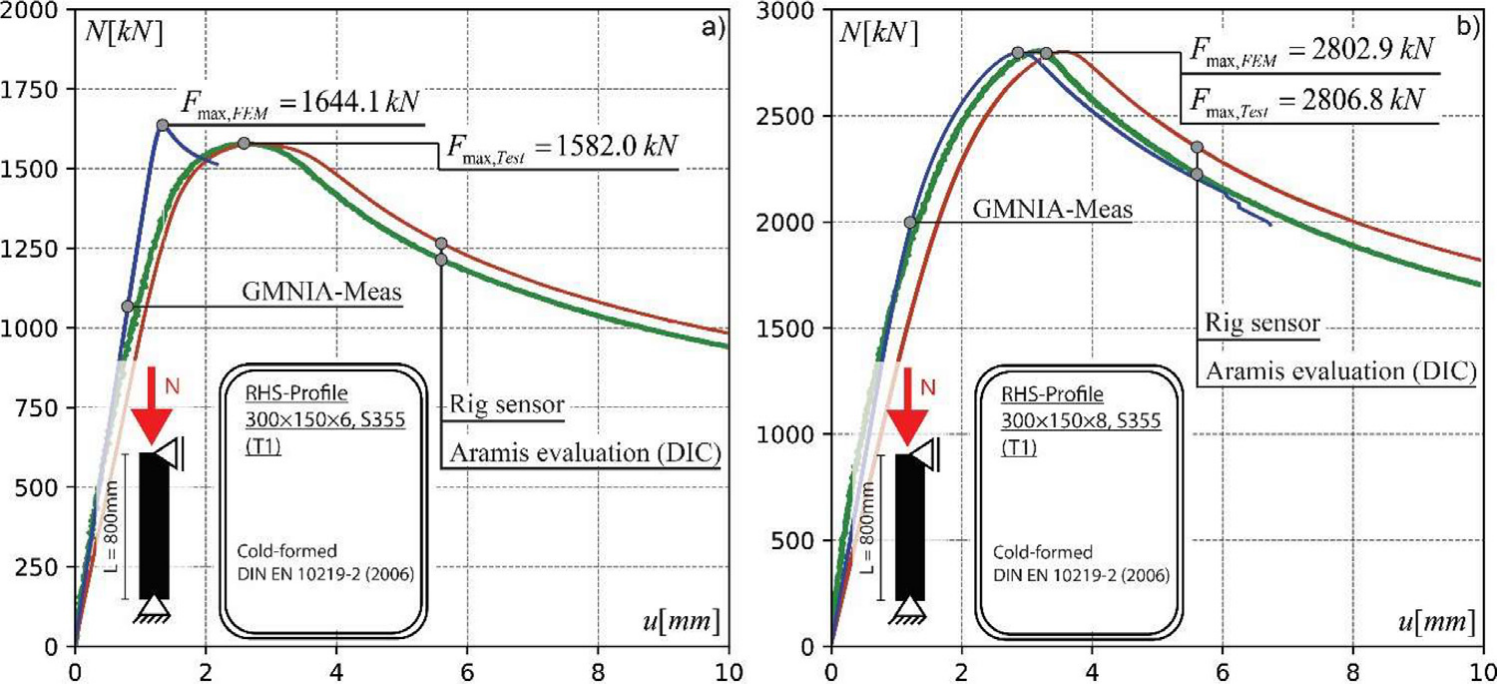
Validation of GMNIA-Meas models against experimental results, adapted from [2].

The following examples shall help to explain the provided data sets. Fig. 1 explains the results of two stub column test for the load case of pure compression. The generated output are three load deformation curves. The red (Rig sensor) and the green curve (Aramis evaluation DIC) are the load-deformation curves from the experiment. This data is directly provided in the folder “Test_Data” within each profile related CSV file. The load–deformation curve represented by the blue line (GMNIA-Meas) is the output from the Abaqus simulation, including the scanned “real” profile geometry. All corresponding data sets are provided in the folder “Simulation_Data” within every profile related Excel file. In addition, the provided Abaqus input (INP) files (see also folder “Simulation_Data”) can directly be considered in own numerical simulations, allowing the user to generate the same load-deformation output. Here, the Abaqus History output with Reaction force “RF1” and Spatial displacement “U1” were used. However, STP files from the folder “Scan_Data” provide additionally the pure 3D geometry and might as well be used in Abaqus CAE, to build an own FE model from scratch.

**Fig. 2:**
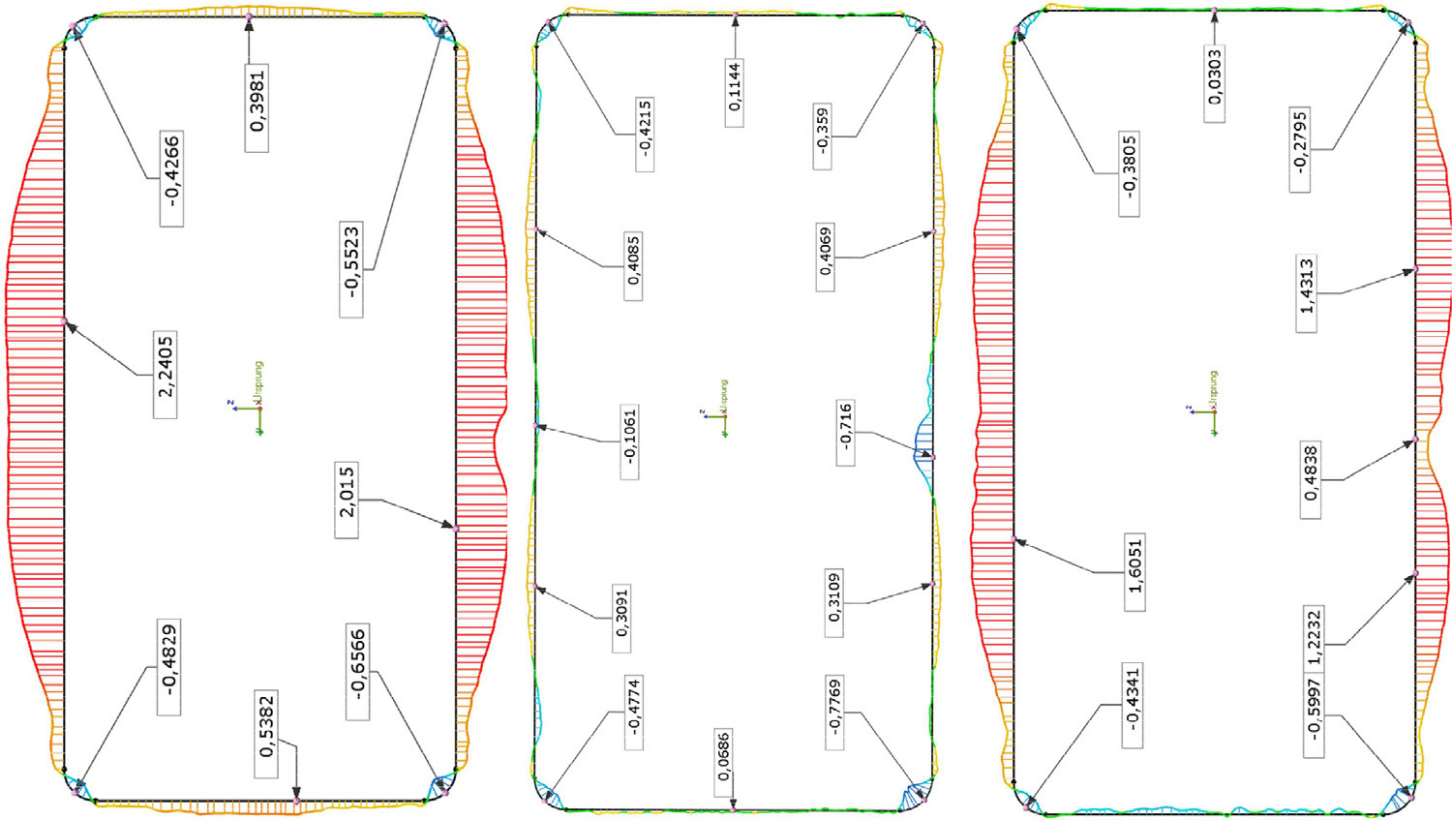
Examplary local imperfection evaluation of an RHS 300 × 150 × 6 profile along the height (h = 800 mm).

The scanned geometry (3D point clouds), provided in the folder “Scan_Data”, can be used for the assessment of local and global imperfections. A possible output of the comparison between the scanned and the perfect geometry (here, according to EN10219-2 [4] for cold-formed steel) is provided in Fig. 2. Circumferential imperfections are compared at three different heights, here done exemplary for an RHS 300 × 150 × 6 profile with the steel grade S355. This specific output was produced with the commercially available software Geomagic Controle X [5].

### 2.1 Experimental Design, Materials and Methods

Full-scale tests are divided into stub-column tests and short beam-column tests. Fig. 4 gives an overview of the chosen setup. The tests were carried out using an MFL Type UPS 1000V test rig. The actuator used for the vertical load transfer is a servo-hydraulic device with a maximum compression force of 10 MN. The actuator is controlled by a servo-hydraulic valve of type Moog 508K03DOJNO D101. A hydraulic load cell is used to measure the actuator force with a pressure transducer of type HBM P3MB-350 bar. All experimental tests were performed displacement controlled, with a displacement ranging from 10.0 mm to 20.0 mm for stub-column tests and from 10.0 mm to 60.0 mm for beam-column tests. The velocity varied between 0.01 mm/s and 0.06 mm/s. A digital image correlation system (DIC) by GOM Metrology was used to capture deformations and rotations of the specimen surface and of the test rig. The reference points and speckle patterns can be seen in Fig. 4a). The DIC recording rate was set to 1 Hz, which provided around 1000 pictures and individual measurements for each specimen. This allowed to capture the overall specimen deformation and the deformation field of the recorded DIC surface. The output of the experimental tests are load-deformation measurements accompanied by DIC deformation measurements as a reference. The overall machine and set-up stiffness was considered and eliminated from the measurements through a calibration test. The positioning of the specimens to introduce different eccentricities for N–M interactions (see Fig. 4b) to d)), was done by using a stiff lever arm with prefabricated positions, i.e. pre-drilled holed in the beam cantilever and in the welded-on top and bottom plates of the test specimens (Fig. 4d)). However, the basic test setup configurations, such as the speckle pattern and its positioning to the camera, as well as the load and displacement measurement of the test rig load cell remained unchanged for all load cases T1 to T5. Test category denominated as “T1” corresponds to stub-column tests with a length of 800 mm, i.e., load case of pure compression. Tests denominated as “T2 – T4” provide an increasing eccentricity to induce an N–M interaction. The specimen length in those cases is equal to 800 mm. Apart from the specimen length, tests denominated as “T5” are in general equivalent to “T3” tests. However, a total length of 2000 mm is used to display an interaction between local and global buckling. An overview of all presented cross-section dimensions, as well as corresponding test categories, is provided in Table 1. The listed eccentricities were measured directly in the laboratory before the tests were carried out.

The outer surface of all specimens was scanned using a Zeiss 3D scanner. The full specimen surface was first recorded as a raw spatial point cloud, including unintentionally scanned areas, e.g., floor surface, marking, etc. Therefore, the scanned data was initially pre-processed in the software “Colin3D” [6] by Carl Zeiss Optotechnik GmbH in order to align individually made scans and make first refinements within the point quality, i.e., removing unnecessary points or filling holes. 3D spline curves were laid over the point cloud using the software “Geomagic Design X” [7], a Computer-Aided Design software controlling the sensitivity and roughness of the spline mesh. The scanned geometry (spatial point clouds) was aligned in space and centered according to the origin of the Cartesian coordinate system in the center of gravity of each profile. Subsequently, this data can either be used to assess geometric imperfections or provide the 3D member geometry in numerical simulations. A reverse engineering workflow is provided in Fig. 5 for both the assessment of local imperfections and the derivation of a finite element model.

The software “Geomagic Control X” [5] was used to make a comparison between shape deviations and tolerances with the nominal geometry according to EN10219-2 [4]. The output of those comparisons is condensed in the provided TXT files. A possible output is described in Fig. 5 (Imperfection Analysis). The output from the software “Geomagic Design X” (STP files) was used as the “real” geometry for the nonlinear FE simulation, so-called geometrically and materially nonlinear analysis with imperfections, here denoted as GMNIA-Meas. A schematic reverse engineering workflow is provided in Fig. 5 (FE Modelling), resulting in a FE-shell model, including the imperfections of the scanned geometry. Linear isotropic shell elements with reduced integration (element type S4R) were considered. These shell elements are formulated with six degrees of freedom associated with each node, three in translation and three in rotation. Each element includes 5 integration points along the shell thickness.

The discretization of the FE mesh in the GMNIA-Meas models depends on the derived spline surfaces from the scanned 3D point clouds. The meshing was directly done in the Abaqus software itself. Therefore, the mesh of each GMNIA-Meas model varies in the total amount of elements, its size, and distribution. The nonlinear material models are based on tensile coupon tests, taken for flat faces and the corners of SHS/RHS profiles. A standard hydraulic testing rig produced by Zwick-Röll was used to perform the corresponding experiments. The outputs of tensile coupon tests (see Table 2) were used for the formulation of non-linear stress strain relations (two-stage Ramberg-Osgood models) based on [8] for cold-formed steel. The engineering stress-strain relations were further transformed into true stress–strain relations for the input in Abaqus. In the following, the material models are directly considered in the Abaqus input files and not explicitly presented in the data sets. Additional information can be found in [2]. Residual stresses were not taken into account. The FEM iteration process was done by using RIKS analysis (iterations based on arc-length control).

**Table 2:** Measured material properties from tensile coupon tests.

Profile	f_y_ in [N/mm^2^]	f_u_ in [N/mm^2^]	ε_u_
RHS 300 × 150 × 6	429	516	14.3
RHS 300 × 150 × 8	451	548	13.9
SHS 140 × 4	430	517	10.8
SHS 200 × 5	401	506	16.0
SHS 200 × 8	475	582	13.2
SHS 200 × 4	563	647	9.6
SHS 200 × 5	557	625	7.0

The boundary conditions were modeled through reference points (RF-Points) located at the top and the bottom edge of the member, which are connected through multiple point constraints (MPC-Beam formulation) to associated node sets along the upper and lower profile outer edge. This boundary condition was used to recalculate the T1, T3, T4 and T5 test. In some cases, a more refined numerical modes was introduced, taking into account the interaction between the test setup parts and specimens, simulating the reciprocal effect of the spherical bearing. An example is shown in Fig. 6.

**Fig. 3:**
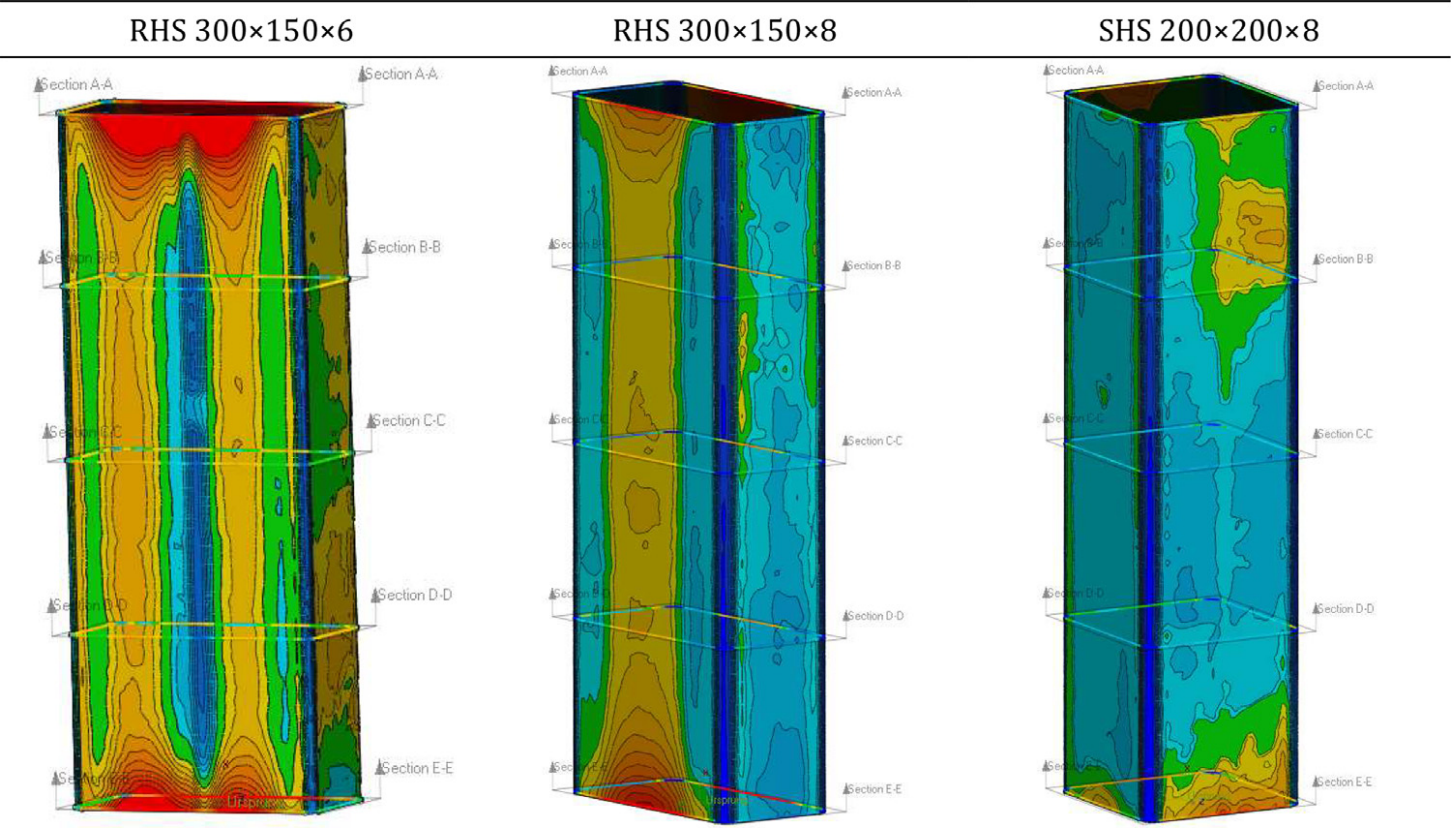
Examplary global imperfection evaluation for several profiles.

Further, T2 tests were performed to investigate the local buckling behavior according to a linear bending moment over the member length. The adapted numerical model was slightly modified compared to the T1, T3, T4, and T5 models. An eccentric load introduction was modeled on the top, as presented in Fig. 6a), explicitly taking into account the spherical bearing through a shell model. A fixed boundary condition (MPC-Beam) was modeled at the bottom to replicate the full-scale experiments (Fig. 3).

**Fig. 4:**
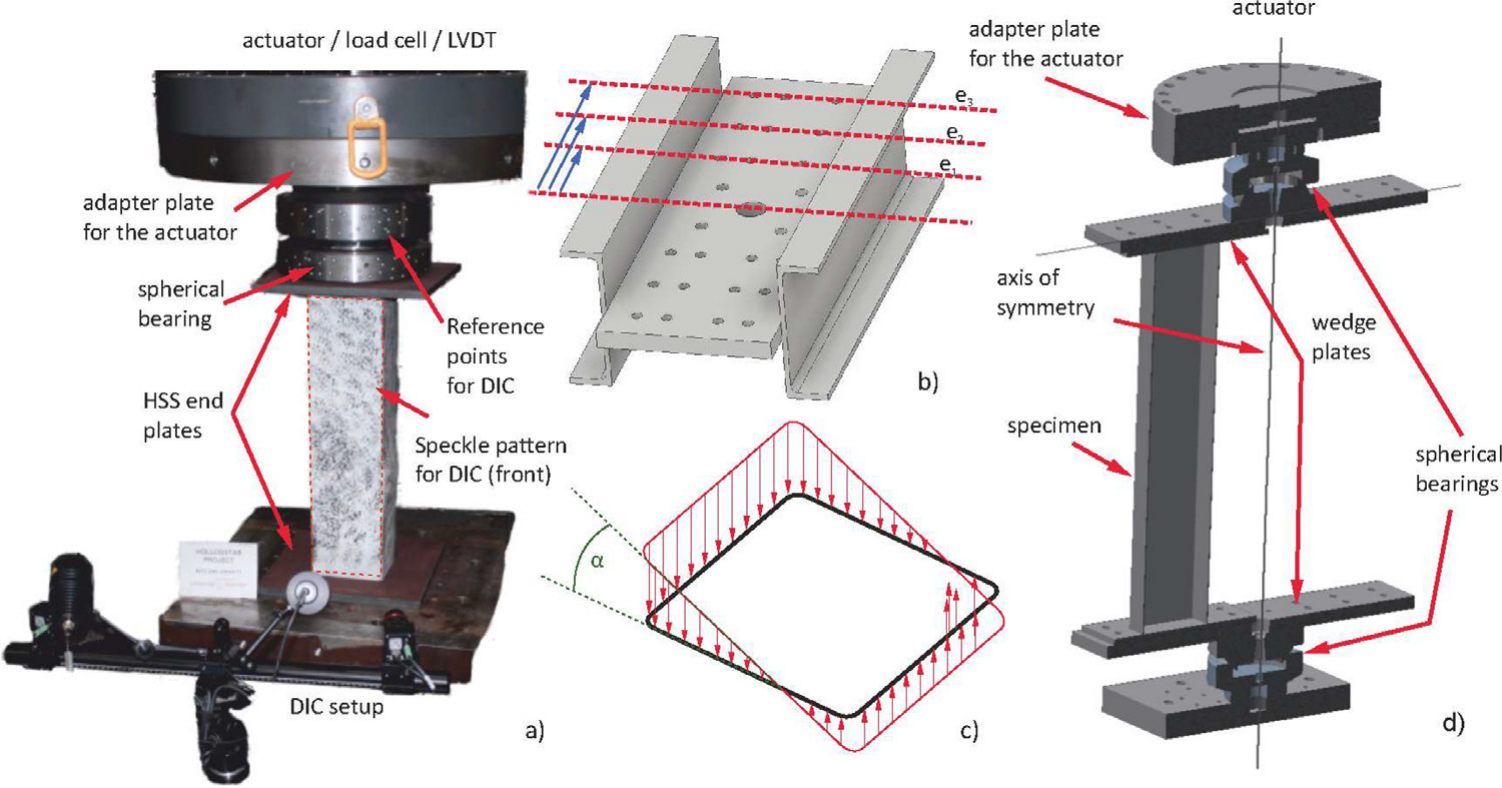
a) Test setup for the stub-column tests; b) eccentricity levels; c) tensional field induced by N+M and corresponding angle of rotation; d) scheme of the N+M test setup.

**Fig. 5:**
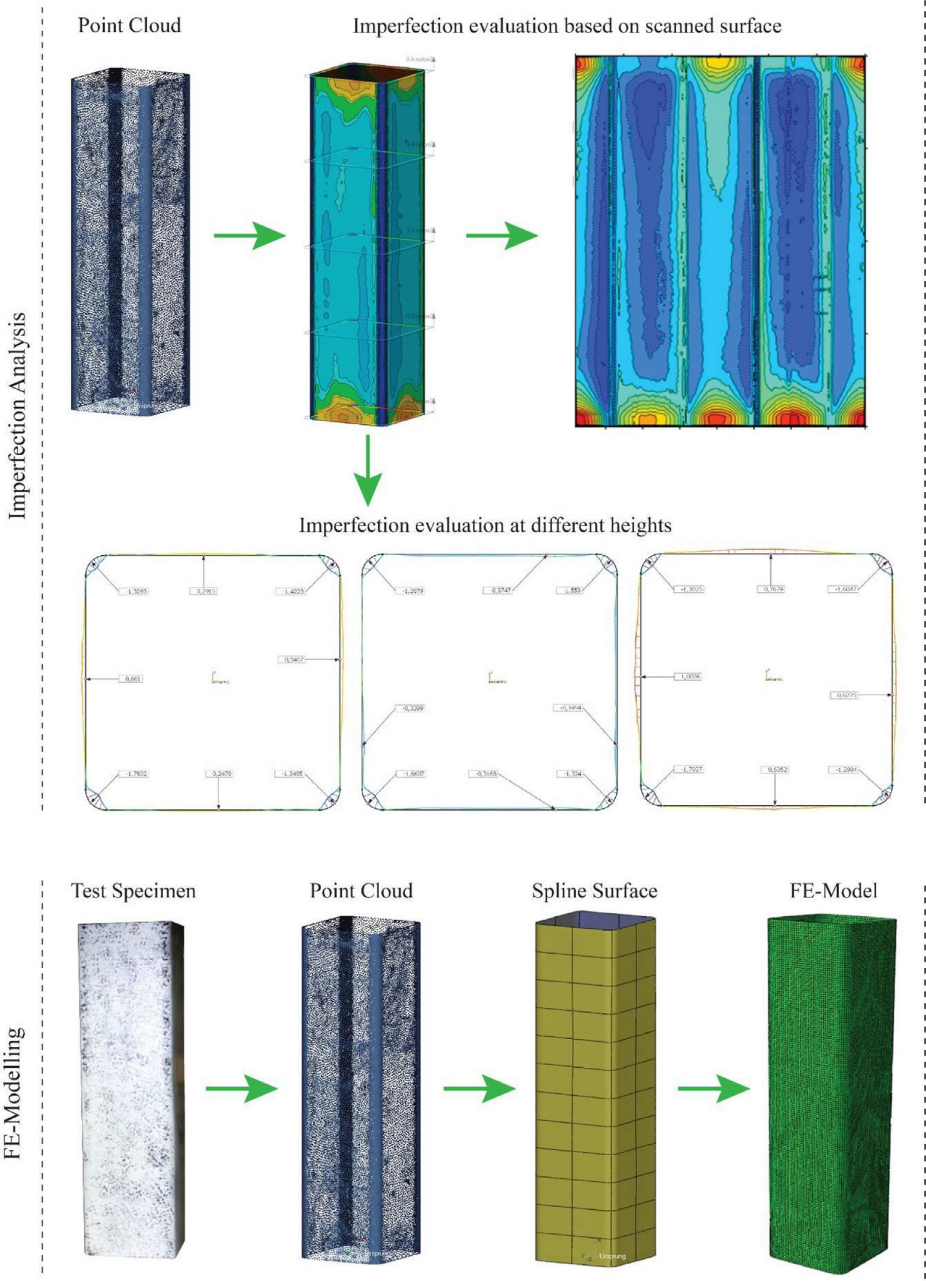
Top) applied workflow for the assessment of local imperfections; Bottom) applied workflow for the use in shell based nonlinear FE simulations (GMNIA-Meas).

**Fig. 6:**
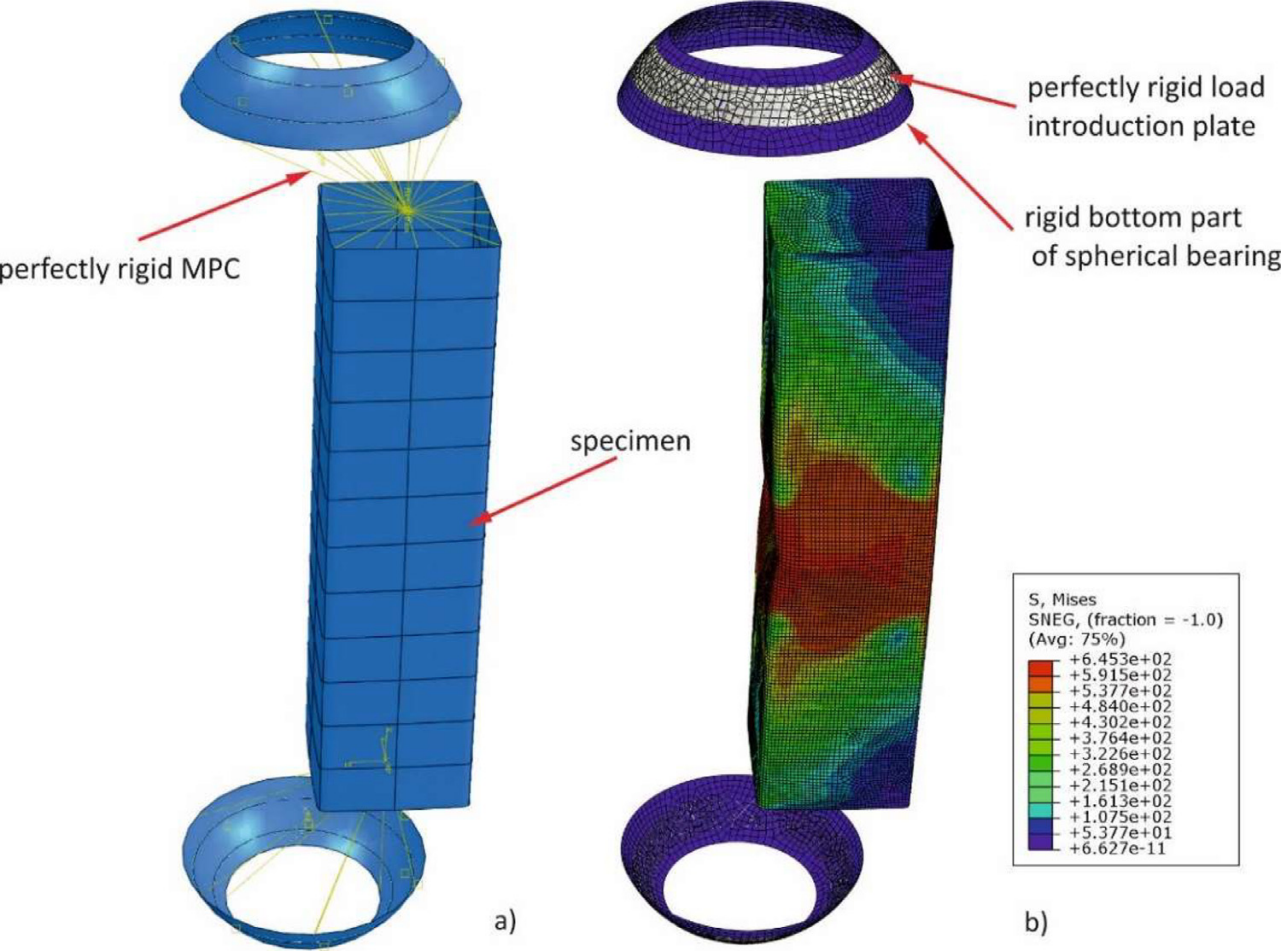
a) Refined FE model with spherical bearing; b) deformed shape and stress results from simulation; based on [2].

## Limitations

• Scanned data from the profile RHS_300_150_6_T2_S355 was unusable. Instead, the scanned geometry from the profile RHS_300_150_6_T1_S355 was used in the GMNIA-Meas simulation.
• Scanned data from the profile SHS_200_8_T2_S355 was unusable. Instead, the scanned geometry from the profile SHS_200_5_T2_S355 was used in the GMNIA-Meas simulation.

## CRediT authorship contribution statement

**Andreas Müller:** Conceptualization, Methodology, Data curation, Visualization. **Andrea Toffolon:** Data curation, Visualization. **Andreas Taras:** Writing – review & editing.

## Data Availability

Hollosstab_SHS_RHS_Data (Original data) (Research Collection).
